# Assessing the Reliability of the Framework for Equitable and Effective Teaching With the Many-Facet Rasch Model

**DOI:** 10.3389/fpsyg.2019.01363

**Published:** 2019-06-14

**Authors:** Priyalatha Govindasamy, Maria del Carmen Salazar, Jessica Lerner, Kathy E. Green

**Affiliations:** ^1^Department of Psychology and Counselling, Faculty of Human Development, Sultan Idris Education University, Tanjong Malim, Malaysia; ^2^Department of Teaching and Learning Sciences, Morgridge College of Education, University of Denver, Denver, CO, United States; ^3^Department of Research Methods and Information Science, Morgridge College of Education, University of Denver, Denver, CO, United States

**Keywords:** many facet, evaluation of teaching, teacher education, Rasch, rater bias

## Abstract

This manuscript reports results of an empirical assessment of a newly developed measure designed to assess apprentice teaching proficiency. In this study, Many Facets Rasch model software was used to evaluate the psychometric quality of the Framework for Equitable and Effective Teaching (FEET), a rater-mediated assessment. The analysis focused on examining variability in (1) supervisor severity in ratings, (2) level of item difficulty, (3) time of assessment, and (4) teacher apprentice proficiency. Added validity evidence showed moderate correlation with self-reports of apprentice teaching. The findings showed support for the FEET as yielding reliable ratings with a need for added rater training.

## Introduction

Teachers in the United States face new levels of accountability due to persistent student achievement and opportunity gaps (Barton, 2005; Boyd et al., 2007; Wagner, 2007; Williams, 2011; Welner and Carter, 2013). Consequently, teacher preparation programs face mounting pressure to prepare effective teachers, particularly for diverse learners (Darling-Hammond, 2009). An emerging body of research indicates that rigorous teacher evaluation increases teacher effectiveness and student achievement (Taylor and Tyler, 2011; Papay, 2012). Increased scrutiny of teacher evaluation has promoted an emphasis on the design of reliable and valid observation-based evaluation models that delineate the competencies of an effective teacher (Daley and Kim, 2010). The purpose of this study was to assess the reliability and validity of a newly developed observation-based measure of K-12 teaching proficiency called the Framework for Equitable and Effective Teaching (FEET). Since the FEET is observation-based, understanding its susceptibility to rater bias is critical. This study was designed to assess rater bias along with FEET item and scale function to find whether the new measure shows promise for general use in evaluation of apprentice teacher competencies. To support more general use, the measure should be easy to use and easy to score – thus while a many facet Rasch model was used to assess measure psychometric quality in this study, the ideal result would show little rater bias and allow item scores to be simply added if the measure is easy to use as a summative evaluation of apprentice teacher competency.

### Theoretical Development of the FEET

Efforts to define equitable and effective teaching have long permeated teacher education reform efforts (Cochran-Smith, 2004; Clarke et al., 2006; Bracey, 2009). The FEET evaluation model emerges from positivist and humanist approaches to defining equitable and effective teaching.

#### A Positivist Approach to Defining Effective Teaching

A positivist approach promulgates a view of teaching based on the development of concrete, observable criteria that result in the enactment of measureable behaviors, or competencies, of effective teaching (Korthagen, 2004). This approach is influenced by behavioral theories of teacher learning developed by John Watson in the early 1900s (Medley, 1979). Current research in teacher evaluation indicates a trend toward behavioral approaches to defining and measuring effective teaching (Korthagen, 2004; Korthagen et al., 2006). A number of contemporary models attempt to provide behavior, or performance-based, definitions of effective teaching, including: the InTASC Model Core Teaching Standards and Learning Progressions (Interstate New Teacher Assessment and Support Consortium, 2013), the National Board of Professional Teaching Standards (), the Marzano Evaluation Model (Marzano Center, 2015), and the Danielson Framework (Teachscape, 2015). The FEET is based on a positivist approach to defining effective teaching in the sense that competencies and indicators are defined, and a rating scale allows for quantitative measurement of proficiency. However, positivist approaches are insufficient. These lack “attention to specific local contexts, human complexity, emotion, and agency” (Sleeter, 2000, pp. 214–215), indicating a need for a humanizing approach to teaching.

#### A Humanist Approach to Defining Equitable Teaching

McGee and Banks (1995) define equitable teaching as “teaching strategies and classroom environments that help students from diverse racial, ethnic, and cultural groups attain the knowledge, skills, and attitudes needed to function effectively within, and help create and perpetuate, a just, humane, and democratic society” (p. 152). Equitable teachers grasp the importance of providing diverse learners with access to values, beliefs, and ways of knowing needed to function in the dominant culture. The FEET incorporates the following: (a) integrate skills for college and career readiness; (b) set high academic expectations; (c) communicate a belief in students’ capacity to achieve at high levels; (d) develop students’ academic language; (e) facilitate the acquisition of content knowledge and skills through discovery, application, and higher-order thinking skills; (f) design units and lessons based on state and national content standards; and (g) implement a classroom management system that facilitates learning.

Diverse learners also need to maintain and develop their cultural resources (Salazar, 2013). The FEET model is infused with culture and prepares teacher candidates to: (a) build relationships with students and parents; (b) engage with communities; (c) incorporate multiple learning styles; (d) engage students in collaborative learning; (e) use instructional strategies to support English language learners and special needs students, (f) incorporate multicultural materials and resources; (g) develop relevant lessons that reflects the cultures of students, counteract stereotypes, and incorporate the histories and contributions of diverse populations; (h) connect content to students’ background experiences, prior knowledge, skills, and/or interests; and (i) and incorporate students’ native language into instruction.

### Instrument Development Procedure

Positivist and humanist theory guided the development of the FEET evaluation model. The FEET includes four dimensions of effective and equitable teaching, with rubrics using a four-level rating scale created with detailed performance indicators. The FEET is used to evaluate pre-service, or apprentice teachers, however, it is applicable to practicing teachers as well. University faculty used the FEET to evaluate pre-service teaching proficiency through classroom observations. The framework includes four teaching dimensions: Engage, Plan, Teach, and Lead. The Plan domain is not part of the observation. Each domain comprises multiple competencies. Raters assign a numerical score for each competency based on the behavior indicators in the rubric. Table 1 shows an excerpt of FEET competency 3.1 and its associated rubric. Each competency has a separate rubric with performance indicators. The FEET development process is described below.

**Table 1:** FEET rubric excerpt.

Competency	Unsatisfactory Indicators (1)	Developing Indicators (2)	Proficient Indicators (3)	Advanced Indicators (4)
3.1 Set context for lesson.	• Delivers lesson without posting, previewing, or reviewing content and language objectives (CLOs).	• Posts content objective only, and/or does not share objective with students during the lesson.	• Posts, previews, and reviews clear, rigorous, measureable content and language objectives (CLOs).	• Engages students in previewing and reviewing standards and content and language objectives (CLOs).
	• Begins lesson without providing a rationale for lesson.	• Shares rationale for lesson that is focused on content knowledge and skills rather than big ideas relevant to students’ lives.	• Provides rationale that connects content to students’ background experiences, prior content knowledge, skills, and/or interests.	• Facilitates student development of the rationale for lesson related to big ideas and essential questions.
	• Lesson is disconnected from real-world application, focusing on rote skills.	• Focuses lesson on content that is missing connections to real-world application, including college and career readiness.	• Promotes real-world application that facilitates college and career readiness.	• Engages students in making real-world connections to the content through their own lenses, and emphasizes college and career readiness.
	• Lacks clarity when communicating performance expectations.	• Communicates performance expectations orally, although expectations are not clearly defined and/or explained in student-friendly language.	• Clearly defines performance expectations orally and in writing using student-friendly language.	• Clearly defines performance expectations and encourages students to provide input into performance expectations.

The first phase of research was completed from 2007 to 2010 through a three-year exploratory qualitative research project. The purpose of the research was to define performance expectations for equitable and effective teaching through the design of a framework for teaching. Frameworks for teaching are commonly used observation-based evaluation models that define, assess, and develop effective teaching (Danielson, 2007; New Teacher Project, 2011). The research question posed in this phase was: What are the dimensions, competencies, and indicators of equitable and effective teaching? This phase included the following procedures: (1) identify performance-based expectations for apprentice teachers; (2) determine the structure and organization of the framework; (3) develop rubrics of performance; and (4) design standardized field-based observation instruments.

First, the researchers identified performance-based expectations for equitable and effective apprentice teachers. The researchers began by analyzing available standards, models, and readiness requirements for apprentice teachers entering the field. The researchers conducted purposeful selection and analysis of public documents related to models, instruments, and research on effective teaching. The data sources included: the Interstate New Teacher Assessment and Support Consortium (InTASC) Model Core Teaching Standards, the National Board for Professional Teaching Standards; two nationally recognized frameworks for teaching (i.e., Danielson Framework and Teach for American Leadership Framework); and 165 peer-reviewed journal articles related to effective and equitable teaching. The articles were selected based on targeted key words in abstracts related to teaching, including: effective, quality, culturally responsive, equitable, multicultural, linguistically responsive, and humanizing. A significant proportion of the articles, 70%, highlighted pedagogical practices that promote the academic achievement of diverse learners by building on their sociocultural resources.

The researchers then analyzed and coded the data through a macro-level deductive content analysis to identify general themes of effective teaching. Subsequently, the researchers used the software, ATLAS.ti, to conduct micro-level inductive content analysis and develop open, axial, and selective coding schemes used to generate themes and sub-themes of equitable and effective teaching (ATLAS.ti, 2015). The emerging data transformation resulted in codes by tallying the number of times concepts occurred in the textual data. This approach revealed key themes and subthemes of effective teaching that recurred across the data sources. The researchers determined how the emerging themes and subthemes would be represented as domains, competencies, and indicators based on degree of specificity. The researchers then conducted an extensive review of the dimensions, competencies, and indicators for alignment, coherence, clarity, appropriate sequence, and practical usage. Next, the researchers compared the data with literature on humanist approaches to defining effective teaching in order to strengthen the focus on equity. Last, the researchers enlisted three faculty members and 10 mentor teachers to establish the content validity of the dimensions, competencies, and indicators. This process helped to establish the FEET’s relevance, representativeness, and accuracy.

Second, once the performance-based expectations were defined, the researchers analyzed the structures of two national frameworks for teaching, the Danielson Framework (2007) and the Teach for America Teaching as Leadership Framework (2015). The frameworks were compared to the emerging FEET dimensions, competencies, and indicators in order to identify strengths and rectify gaps in the FEET, and provide a template for the structure and organization of the Framework. The FEET is structured in a way that moves from the simple themes related to equitable and effective teaching (e.g., dimensions), to more detailed descriptions of performances (e.g., competencies), and evidence of behaviors indicating the performances are evident (e.g., indicators).

Third, once the researchers identified performance-based expectations for apprentice teachers, and determined the structure and organization of the framework, the next step was the development of rubrics of performance. Numerical rating scales are often used to quantify observations resulting in greater accuracy and objectivity of observational reports (Milanowski, 2011). The rubrics provide exemplars of performance at four levels of proficiency.

Last, after developing the rubrics, the researchers developed an observation instrument to facilitate the practical implementation of the FEET, and to allow for summative and formative assessments of apprentice teachers. Raters use the rubrics to provide a quantitative assessment of apprentice teacher performance. The observation instrument is intended to be utilized by experts, or supervisors, in the field. These supervisors have the experience and understanding of the content skills and knowledge to judge an apprentice teacher’s mastery level. They are raters or judges and they play a central role in rater-mediated assessments. But, raters can contribute undesired variance in ratings (Farrokhi et al., 2011). If the variability contributed by raters is substantial it manifests in various forms of rater errors and is referred to as construct irrelevant variance (Downing, 2005). Although these rater errors are irrelevant to the construct, they affect ratees’ performance scores. Raters can vary in terms of the severity/leniency in their ratings, consistency in ratings, and can display biases on items, subjects, or rating categories (Farrokhi et al., 2011). These different sources of variability can be collectively addressed as rater error or rater effects.

The purpose of this study was to assess the psychometric quality of the FEET. The FEET was completed by the apprentice teachers’ supervisors twice per quarter during their first year of coursework and student teaching. Supervisors’ ratings were analyzed in this multi-faceted study. The intent of this work was to provide direction for student supervisor training and for item revision of the instrument. Variability in (1) rater judgments, (2) item difficulty, (3) time of assessment and (4) apprentice’s proficiency levels were evaluated.

The research questions that directed this study were:

(1) Do the items vary sufficiently in difficulty?
(2) Do supervisors differ in the severity or leniency with which they rate teacher apprentice performance in teaching?
(3) Do supervisors exhibit bias when using the items in the instrument?
(4) Is progression over time seen with use of the measure?
(5) Does the instrument provide evidence of convergent validity?

## Materials and Methods

### Participants: Apprentices and Supervisors

#### Participants

The participants in this research project included eight TEP supervisors and 59 teacher apprentices. Of the eight supervisors, or raters, four were appointed faculty members, and four were adjunct faculty. The supervisors’ areas of teaching and research expertise included: urban education, cultural and linguistic diversity, bilingual education, teacher evaluation and coaching, aesthetics, and teacher renewal. Seven of the eight supervisors were White and one was Latina. Seven of the supervisors were female. The supervisors all had 3–5 years of experience evaluating teacher candidates. They had a combined expertise of 77 years teaching in K-12 schools. Two of the supervisors held doctoral degrees in education, three were doctoral candidates in education, and three held master’s degrees in education.

Of the 59 teacher apprentices, 23% of the students self-identified as students of color. Male participation was 38%.

### Instrument

The FEET, described in detail above, comprises 11 items and all the items were measured using a 4-point rating scale with categories 1 = unsatisfactory, 2 = developing, 3 = proficient, and 4 = advanced. See Supplementary Appendix A for the FEET measure. The FEET administration data summarized herein are from the 2015–2016 academic year.

Two validation measures were administered at the end of the program using one statewide measure and a local measure created specifically for the teacher preparation program. The instruments were the Core Competencies of Novice Teachers Survey (Seidel et al., 2011) and the Teacher Education Program Satisfaction Survey (2015).

### Core Competency Survey (CCS)

The *Core Competency Survey* (CCS) (Seidel et al., 2011) was administered to teacher program graduates as a self-report of teaching competencies. The instrument contains 46 statements related to eight core competencies related to effective teaching: (1) demonstrating mastery of and pedagogical expertise in content taught; (2) managing the classroom environment to facilitate learning for students; (3) developing a safe, respectful environment for a diverse population of students; (4) planning and providing effective instruction; (5) designing and adapting assessments, curriculum and instruction; (6) engaging students in higher order thinking and expectations; (7) supporting academic language development and English language acquisition; and (8) reflection and professional growth. The response scale asks participants to report how well prepared they are by their teacher education program on a 1–4 response scale with 1 = not well prepared and 4 = very well prepared. Exploratory factor analysis of the development sample yielded a dominant first factor with Cronbach’s alpha for the total score exceeding 0.85 (Seidel et al., 2011). The total score was used in this study.

### Teacher Education Preparation Satisfaction Survey (TPS)

Additionally, the *TEP Satisfaction Survey* (TPS) was specifically oriented to the University of Denver’s Teacher Education Program. The TEP Satisfaction Survey (2015) assesses self-reported proficiency based on coursework. The 46 items relevant to candidate performance are comprised of a 22-item subscale asking for self-reported competence related to fieldwork, a 22-item subscale asking for competence related to coursework, a single item global self-rating of overall teaching competence, and a single-item related to how the candidate thought the field supervisor would rate him/her.

Two adjunct faculty members and one appointed faculty member were selected to conduct an expert review of the survey content in order to establish the content validity of the survey. Each of the reviewers was a current TEP supervisor and was familiar with all aspects of TEP and the FEET. Expert reviewers assessed each survey item for relevance, difficulty, and clarity. The expert review indicated survey items were generally low in difficulty, high in relevance, and high in clarity. Reliabilities for the two multi-item subscales were 0.95 and 0.96.

### Procedure

Supervisors were assigned to conduct two observations of non-supervisees per quarter, for a total of six observations throughout the 3-quarter instructional sequence. This was in addition to observations of pre-assigned supervisees per quarter, for a total of six additional observations. In total, each supervisor completed 12 observations of teacher candidates for the 2015–2016 academic year. More than one supervisor rated each candidate in a rating scheme designed to ensure connectivity or linkage – that is, all supervisors overlapped in rating apprentices so the data were connected. Linkage is needed between all elements of all facets so that all parameters can be estimated without indeterminacy within one frame of reference. In the rating scheme used, connectivity was adequate and there were no disconnected subsets. Observations were scheduled jointly by the supervisor and teacher candidate and occurred throughout the academic quarter at the teacher candidate’s school site. Observations occurred in K-12 classroom settings and took on average of 45–60 min.

The researchers designed and implemented standardized protocols and training for supervisors in order to minimize rater bias. This included the following: (a) design training protocols, including procedures, training manual, and benchmark and rangefinder videos used for scoring practice; (b) delineate protocols for candidate observations; (c) establish scoring parameters or guidelines; and (d) develop a standardized training for supervisors. The supervisors participated in the first FEET evaluation training in September of 2015.

The researchers correlated candidate position on the FEET with scores from the two validation measures in order to establish convergent validity estimates. Validation measures were administered via an on-line survey given in spring 2016.

### Analysis

The Many-Facet Rasch Model (MFRM) was used to model the variability and bias in ratings. The variance in ratings that can be attributed to raters and other sources can be compared. The MFRM is an extension of the Rasch model which simultaneously calibrates all rating facets in a single common scale that can be used to estimate a person’s score. The MRFM not only allows monitoring the effects of differing severity of raters but offers adjustments for systematic rater error in the ratee final score (Downing, 2005). The MFRM can also help to determine whether raters are exhibiting effects besides severity (Myford and Wolfe, 2003, 2004). The bias analysis in MFRM uncovers the interaction between the rater and other facets in the rating schema. While the MFRM allows apprentice measures to be corrected for facets such as rater bias, measurement occasion, and item difficulty, the focus of the present study was to assess the impact of those facets, in particular rater bias, rather than to generate corrected scores for apprentices. That is, apprentice scores corrected for other facets in the design were not used to inform apprentice grades in this study.

An MFRM was used to evaluate the apprentice teacher performance over 1 year of coursework using the FEET. In this study, a four facet Rasch model was used. The facets were: (1) apprentice, (2) item, (3) supervisor, and (4) evaluation occasion (time). The probability of an apprentice (*n*) with competence (*B*) obtaining a rating of *x* (*x* = 1, 2, 3, 4) on item *D* from supervisor C with item category difficulty *F* at time *T* (*t* = 1, 2,…,6) is expressed as the following:

$$\text{Log}\;(P_{nijkl}/P_{nij\left(k-1\right)l})\;=\;B_{n}\;-\;D_{i}\;-\;C_{j}\;-\;F_{k}\;-\;T_{l}\;\;\;\;\;\;\;\;\;\;\;\;\;(1)$$

The performance on each facet on the rating responses was evaluated. Empirical indices were examined for each facet to ensure that the facets were performing as intended. The assessment of individual facets helps to provide direct facet-related feedback for improvement. In total, there were eight raters assessing 59 apprentices on 11 items over 6 occasions. The FACETS (version 3.71.2, Linacre, 2015) software was used to analyse the four-facet model. Chi-square tests, fit indices, separation ratio, and reliability of separation indicators were used to determine the performance of each individual facet. All of the statistical indicators were examined for each facet. These statistical indicators were used as evidence to draw conclusions about the quality and deficiencies of the instrument.

The chi-square tests for facets, facet measures (logit), facet separation ratio, and the facet reliability indicators were examined to understand dispersion and fit of elements in a facet (e.g., raters, items). The fixed chi-square statistic (fixed effects) provides information about the heterogeneity/difference between the elements of a facet. A statistically significant chi-square result rejects the null hypothesis that elements are at the same position. The random chi-square statistic provides information about whether facet elements can be regarded as a random sample from a normal distribution. If non-significant, elements can be regarded as coming from a normal distribution sample.

Separation gives the spread of the facet measures relative to the precision of those measures. Elements are similar to each other in terms of their position if this value is closer to zero. This index helps to determine if the differences are larger than random measurement error. A higher separation ratio (*G_j_*) shows greater spread of the measures.

Unlike internal consistency reliability, higher “reliability” represents greater variability among the raters/supervisors. Reliability here is the variance of the rater severity measure over the measurement error. Greater variance of the rater severity indicates the presence of variability among the raters. For the remaining three facets, reliabilities is interpreted similarly to interpretation of Cronbach’s alpha.

Researchers generated a variable map that presents the position of all the facets in a single layout, also known as a Wright map. This is used to represent the calibration (position) of each facet in the analysis; thus, the researcher is able to make visual comparisons within and between various facets, and gain an overall understanding of the measure. The first column represents the range of the measure in logits. The facets were set to be negatively oriented except for the candidate facet. Therefore, supervisor, time, and items with negative measure means that the supervisors are lenient, candidates are rated lower, and items administered are easier. The positive measures identify supervisors who are more severe raters, candidates with higher ratings, and more difficult items. The second column corresponds to supervisor severity or leniency exercised when rating the apprentice. The third and fourth columns present the identification number assigned to the candidate and the distribution of the candidate related to teaching skill proficiency. The fifth column displays candidate proficiency across time of evaluations. The sixth column indicates the item difficulty. The variable map presents a visual representation of the individual facets and the associations between the facets.

## Results

The variance explained by the Rasch measure, an indicator of dimensionality of the item set, was 41.75%, suggesting a unidimensional construct underlying the 11 FEET items. The response scale was used appropriately, with no inversions in Rasch-Andrich thresholds or observed average, though response option 1 (unsatisfactory) was used only 1% of the time. Table 2 lists the scale function indices.

**Table 2:** Scale use indices.

			Rasch-Andrich	Average
Score	Count	Percent (%)	Threshold	Measure
1	30	1	–	–1.91
2	787	28	–4.57	–0.28
3	1762	62	–0.03	1.76
4	251	9	4.60	3.47

### Item Facet

**Figure 1:**
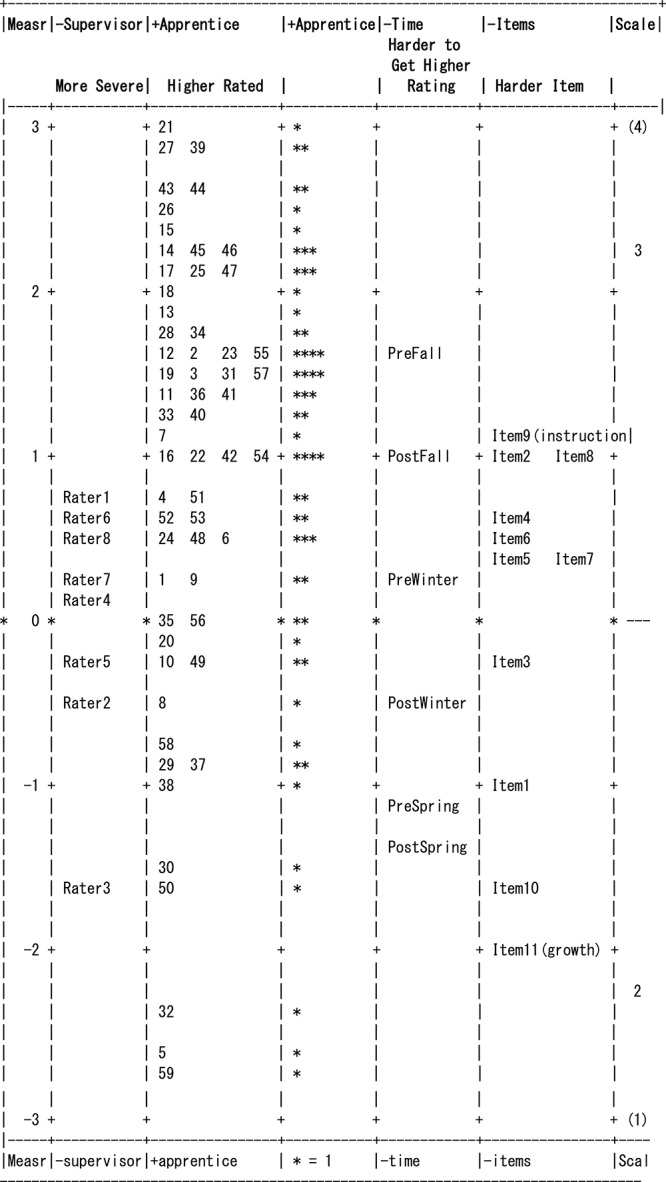
Item-person map.

The chi-square test statistic for items, *X*^2^(10) = 731.6, *p* < 0.001, indicated significant differences in the item difficulties The fit statistics identified all except one of the 11 item’s mean square values as fitting within a range of 0.5 to 1.5 and so were *productive of measurement* (Linacre, 2013). Item 6 (rigorous academic talk) evidenced some misfit and would be a candidate for revision or added supervisor training. An item separation ratio of 6.45 shows the variability between the administered items. The logit measure of item difficulty ranged from a low of –1.95 (easy item) to a high of 1.06 (difficult item). Item reliability of separation (0.98) supports existence of variability in level of difficulty among the items. FEET shows the ability to identify and distinguish different levels of proficiency. Although FEET items ranged along the proficiency continuum they were generally clustered at mid-range. Thus, the items are not spread out along the entire difficulty continuum, with a range of approximately 3 logits. In general, the items need to be reviewed again to ensure that the different levels of teaching skills proficiencies are well-represented. Table 3 provides the sample RMSE, separation, reliability, and results of the fixed and random chi-square tests for this and the other facets. Table 4 presents item difficulty measures, standard error of the measures, and infit and outfit mean squares. Figure 1 is the Wright map and presents positions of all facet elements.

**Table 3:** RMSE, separation, reliability of separation, and fixed and random chi-square tests by facet.

	Item	Supervisor	Time	Apprentice
RMSE	0.16	0.12	0.10	0.34
Separation	6.45	5.87	10.54	3.91
Reliability of Separation	0.98	0.97	0.99	0.94
Fixed Chi-square	*p* < 0.001	*p* < 0.001	*p* < 0.001	*p* < 0.001
Random Chi-square	*p* = 0.36	*p* = 0.34	*p* = 0.29	*p* = 0.57

**Table 4:** Item difficulty measure, standard error, and fit indices.

			Infit	Outfit
	Measure	SE	Mean	Mean
Item	(logit)	(logit)	Square	Square
11-Demonstrate growth	-1.95	0.12	0.72	0.70
10-Meet professional standards	-1.64	0.12	0.75	0.72
1-Develop respectful relationships	-1.04	0.12	1.03	1.01
3-Actively engage students	-0.30	0.12	1.13	1.17
7-Make content and language	0.34	0.23	0.60	0.57
comprehensible
5- Facilitate rigorous learning	0.36	0.15	1.28	1.30
6-Rigorous academic talk	0.55	0.23	1.62	1.66
4-Set context for lesson	0.59	0.12	1.09	1.11
2-Equitable classroom management	0.97	0.12	1.06	1.07
8-Use formal and informal assessment	1.05	0.12	0.97	0.98
9-Differentiate instruction	1.06	0.22	0.88	0.88

*SE = Standard error*.

### Supervisor Facet

The fit statistics identified all eight supervisors’ mean square values as fitting within the range of 0.5 to 1.5. The fixed chi-square, *X*^2^(7) = 270.3, *p* < 0.001, showed that the supervisors’ severity ratings were significantly different. The Rasch-kappa (Eckes, 2015) was nearly zero (κ = 0.01). Findings from the chi-square test indicate that the supervisors did not have the same level of severity/leniency in evaluating the apprentices. The supervisor’s reliability of separation (0.97) supports the presence of distinctive levels of severity/leniency among the sample of supervisors. The logit measure of supervisor severity ranged from a low of –1.57 (lenient supervisor) to a high of 0.80 (severe supervisor) (see Table 5). But, a closer evaluation of the levels of severity/leniency showed rater’s logit position as not far from each other except for Rater 3. Since the raters showed significant differences in their logit position, the difference in the levels of severity/leniency is considered a call for further rater training in this context, especially for Rater 3.

**Table 5:** Summary of supervisor measure and fit statistics.

	Measure	SE	Infit Mean	Outfit Mean
Supervisor	(logit)	(logit)	Square	Square
Rater 3	-1.57	0.13	1.07	1.09
Rater 2	-0.50	0.11	0.92	0.92
Rater 5	-0.23	0.11	1.17	1.19
Rater 4	0.16	0.13	0.92	0.91
Rater 7	0.23	0.11	0.81	0.79
Rater 8	0.55	0.16	0.96	0.97
Rater 6	0.56	0.10	1.12	1.13
Rater 1	0.80	0.10	0.95	0.94

*SE = Standard error*.

### Time Facet

The fit statistics identified all time ratings as fitting, or perhaps fitting too well for the fall observations (with fit indices < 0.50). The fixed chi-square, *X*^2^(7) = 689.5, *p* < 0.001, showed that the ratings were significantly different over time. The logit measure by time ranged from a low of –1.35 (post-test in spring) to a high of 1.17 (post-test in fall) (see Table 6). The difference in performance scores, which generally increased from the first observation in fall quarter to the last observation in spring quarter, supported the notion that (1) apprentice performance improved over the course of the year, (2) apprentices learned what observers were looking for in their performance, or (3) observers expected better performance with time and were more familiar with both the FEET tool and the apprentices.

**Table 6:** Summary of time measure and fit statistics.

	Measure	SE	Infit Mean	Outfit Mean
Time	(logit)	(logit)	Square	Square
Pretest-Fall	1.57	0.10	0.83	0.83
Posttest-Fall	1.05	0.12	0.83	0.82
Pretest-Winter	0.27	0.10	1.05	1.06
Posttest-Winter	-0.46	0.09	1.09	1.08
Pretest-Spring	-1.08	0.10	1.05	1.06
Posttest-Spring	-1.35	0.09	1.06	1.06

*SE = Standard error*.

### Apprentice Facet

The fit statistics identified all except one apprentice as fitting within the range of mean square fit from 0.5 to 1.5. The fixed chi-square, *X*^2^(7) = 903.1, *p* < 0.001, showed that the performance ratings differed across apprentices. The reliability of separation (0.94) supports the presence of distinct levels of performance among the sample of apprentices. The logit measure of apprentice performance ranged from a low of –2.77 (least proficient) to a high of 2.94 (most proficient). This difference in apprentice logit positions reflects raters’ ability to use the items to distinguish among apprentices’ teaching skills proficiency.

### Rater by Item Interaction

The objective of the bias-interaction analysis was to determine if some supervisors had specific biases for some of the items. A statistically non-significant chi-square, *X*^2^(88) = 108.6, *p* > 0.05 indicates that raters did not differ significantly overall in using the items. The item ratings were generally invariant across the raters though there were some significant bias-interactions that explained a total of 3.93% of residual variance. The finding helps to support the quality of the items and ratings in the instrument.

### Validity Assessment

The Pearson correlations between scores on the FEET measure, CCS, and the TPS were calculated and are presented in Table 7. Visual inspection of scatterplots showed no evidence of non-linearity. The highest correlation (*r* = 0.43, *p* < 0.01) was found between the FEET score and how students perceived they would be rated by their field supervisor. The only other statistically significant correlation was between self-reported global performance and FEET score (*r* = 0.37, *p* < 0.01). How well-trained students perceived themselves to be as reported on the CCS was not related significantly to observer ratings of performance. These results suggest self-perceptions of teaching proficiency are statistically significantly but not strongly related to supervisor perceptions of teaching proficiency.

**Table 7:** Correlations between FEET and TPS and CC scales.

Instrument used for convergent validity	FEET
**Teacher Performance Survey subscales**	
TPS Field	0.25
TPS Course	0.21
TPS Global	0.37**
TPS Field Supervisor Rating	0.43**
**Core Competency Survey**	
CCS total score	0.19

*^∗∗^p < 0.01*.

In summary and in response to the research questions, items varied in difficulty though the construct coverage could be improved, supervisors varied significantly in rating severity though little overall bias (supervisor–item interaction) was shown, higher ratings were shown over time as apprentices progressed through the program, and some evidence of convergent validity was found though FEET ratings were clearly not strongly related to apprentice self-reports of competencies.

## Discussion

The results from item fit indices, severity/leniency of the raters, and the interaction between the raters and the items were used to assess the quality of the FEET instrument. In terms of the items, the 11 items covered a 3-logit range. One misfitting item was detected in the analysis. Bias analysis indicates that the items were generally invariant across the raters with approximately 4% of the variance explained by bias (rater/item interaction) terms. The findings from item, rater, and the interaction between the rater and items analyses showed support for the FEET as yielding reliable ratings.

The objective of this research project was to investigate the psychometric properties of the FEET (e.g., scale use, fit, consistency, convergent validity); and identify implications for revising the FEET evaluation model and its effectiveness to train supervisors to evaluate apprentice teacher competencies. Overall, the supervisor, apprentice, time, and item facet analysis indicate that the FEET has adequate measurement quality, with apprentices progressing over time. Ratings of apprentices improved by nearly three logits – a substantial change – over the course of the program, in a coherent progression, suggesting competencies were gained over the course of the year and, more importantly for the purpose of this manuscript, were reflected by the measure. The supervisors showed a good understanding and use of the FEET evaluation instrument. There was no randomness in the way the supervisors assigned the ratings. The supervisors also showed evidence of distinguishing the apprentices’ abilities and rating them at different performance levels. While the supervisor ratings were fitting, they also had significant differences in the severity of the apprentice ratings. The variability in supervisor ratings indicates a need for improved supervisor training; this may include the use of range finder videos for practice scoring, a review of scoring rubrics, and frame of reference training to an agreement criterion. If the FEET is implemented broadly and scores are used for summative purposes, it is critical to train raters to a criterion *or* to continue use of an MFRM analysis so that rater bias can be controlled in obtaining apprentice final scores. If rating severity or leniency is a trait for a particular rater, it may be difficult to remediate.

The apprentices’ ratings were similar to the ratings expected from the model. This suggests minimal error from the apprentice facet to the measurement model. This indicates that the majority of apprentices demonstrated proficiency in the development of teaching skills, as rated by the FEET. Moreover, their teaching proficiency increased over time. Two students were overfitting, indicating that supervisors may be overestimating or underestimating the skills of some students. Moreover, the separation reliability of items was adequate although there were few items with intermediate levels of difficulty. It is suggested that the items or scale response options potentially be revised to obtain a more diverse spread of item difficulty levels.

Last, while some evidence of convergence with external measures (CCS, TPS) was found, it was clear that self-perceptions were not strongly related to supervisor perceptions of teaching proficiency. This result is similar to those found in other content areas where there is potential for the influence of perception and also clarity in construct definition that differs from self-report to observation (e.g., Hites and Lundervold, 2013).

The results of the study indicate that supervisors showed adequate reliability and the FEET demonstrated adequate measurement quality, thereby indicating the success of the FEET evaluation model in assessing apprentice teacher proficiency. The results also point to specific areas of improvement for the supervisor training and FEET evaluation model, including: (a) improve supervisor training through the review of FEET rubrics and the use of a range finder video to decrease the variability of ratings among supervisors; (b) provide individual training to the most severe and most lenient supervisors; (c) examine the FEET item difficulty progression and potentially revise one item; (e) analyze the data on overfitting students to see if there are patterns or contextual factors that may have impacted apprentice ratings. At this point in its development, results suggest that the FEET may be useful for formative evaluation but for summative purposes, apprentice scores would need to be adjusted, particularly for rater severity through use of a MFRM.

Future research initiatives include a second MFRM study, replication with a larger number of supervisors, and the completion of a project in which the researchers estimate the predictive validity of the FEET evaluation measure by comparing pre-service teacher summative evaluation ratings to their in-service teacher effectiveness ratings and student outcomes. This research and future research are important because the FEET can be used to prepare apprentice teachers or develop practicing teachers. Initial research on the FEET demonstrates that this model shows support for reliability and validity in the preparation and development of effective and equitable teachers.

## Ethics Statement

This study was carried out in accordance with the recommendations of the Office of Research Integrity and Education, Human Research Protection Program. The protocol was approved by the Institutional Review Board, University of Denver. Written, informed consent was obtained from all observers and apprentice teachers who served as participants in the study.

## Author Contributions

PG consulted on data collection, conducted analyses, and wrote portions of the manuscript. MdCS obtained funding to support the project, conducted the research to develop the measure, and wrote portions of the manuscript. KG supervised the data collection, consulted on analyses, and wrote portions of the manuscript. JL participated in measure development research and wrote portions of the manuscript.

## Conflict of Interest Statement

The authors declare that the research was conducted in the absence of any commercial or financial relationships that could be construed as a potential conflict of interest.
