# Supplementary material for: Assessing the Reliability of the Framework for Equitable and Effective Teaching With the Many-Facet Rasch Model
Source: Front Psychol. 2019 Jun 14;10:1363. doi: 10.3389/fpsyg.2019.01363 (PMC6587337; doi:10.3389/fpsyg.2019.01363)
Supplement: Supplementary file 1 [file Data_Sheet_1.docx]

Appendix A: FEET Dimensions, Competencies, & Indicators

| DIMENSION 1: **ENGAGE** students in an inclusive and supportive learning community.  **1.1 Establish respectful and productive relationships with students and families (Item 1).**  E.1 Demonstrates interest, value, and respect for students’ family members, home cultures, and communities.  E.2 Demonstrates positive rapport with students and facilitates positive rapport between students (e.g., empathy, patience, caring).  E.3 Communicates belief in capacity of all learners to achieve at high levels (e.g., college and career readiness, high expectations).  E.4 Communicates with parents/families to gather information on student needs, provide support, and share data about student progress.  **1.2 Use equitable classroom management strategies (Item 2)**  E.5 Implements a developmentally appropriate, predictable, and proactive behavior management system that promotes student accountability (e.g., precise directions, positive narration, rewards/consequences).  E.5 Ensures students follow behavior norms by promptly redirecting inappropriate behaviors, and enforcing consequences  E.7 Uses predictable transition strategies effectively to maximize time on task.  E.8 Uses an efficient process to ensure students have appropriate materials for learning.  E.9 Incorporates student voice and choice in developing classroom community.  **1.3 Actively engage students in learning (Item 3)**.  E.10 Uses a variety of active engagement strategies (e.g., interactive technology, manipulatives, realia, total participation techniques) to ensure all students participate.  E.11 Provides learning experiences that target multiple learning styles, including auditory, visual, kinesthetic, tactile, and social/interpersonal.  E.12 Provides opportunities for students to experience joyful learning that includes discovery, application, and/or collaboration. |
| --- |
| DIMENSION 2: **PLAN** rigorous and relevant, standards- and outcome-based lesson and unit plans.^a^  **2.1 Use backward design curriculum planning to develop units of study.**  P.1 Identifies big ideas, essential content concepts, and enduring understandings.  P.2 Creates logical and connected units of study that are aligned to relevant content and language standards.  P.3 Uses, modifies, supplements, or adjusts district-approved curriculum to plan standards-based learning goals and adapt curriculum to students’ needs.  P.4 Includes materials and resources that reflect the culture(s) of students and include a variety of cultures.  P.5 Designs rigorous and relevant unit performance tasks.  P.6 Develops a sequence of lessons aligned to unit goals.  **2.2 Design measureable, challenging, and relevant lessons**  P.7 Sets clear, rigorous content and language objectives based on unit goals and measurable learning outcomes.  P.8 Creates a logical sequence in lesson plan, with each component aligning to lesson objectives and assessment methods.  P.9 Designs lesson experiences that require students to use higher-order thinking strategies (e.g., including analyzing data, thinking creatively, developing and testing innovative ideas, problem solving, synthesizing knowledge, and evaluating conclusions).  P.10 Draws on student diversity (e.g. race, ethnicity, gender, abilities, sexual orientation, religion, culture) to design lessons that reflect the culture(s) of students, counteract stereotypes, and incorporate the histories and contributions of diverse populations.  **2.3 Analyze and develop assessments and use data to plan instruction.**  P.11 Analyzes assessments for validity, reliability, and/or bias.  P.12 Develops various formative and summative assessment tools to gather comprehensive data on students’ knowledge and skills.  P.13 Uses student assessment data to set SMART goals.  P.14 Uses assessment data to design differentiated learning experiences for diverse learners (e.g. ELL, special education, gifted).  P.15 Uses assessment data to analyze trends in student progress, identify strengths and needs, and set individual learning goals.  **2.4 Demonstrate knowledge of content and student development.**  P.16 Analyzes relevant content standards and current research on content pedagogy to identify implications on student learning.  P.17 Understands how students’ cognitive development impacts content learning.  P.18 Anticipates prerequisite content and language knowledge and skills including typical errors, misconceptions, and difficulties.  P.19 Uses knowledge of content to plan rigorous and relevant units and lessons that develop academic language, literacy, and numeracy. |
| DIMENSION 3: **TEACH** equitably by establishing high expectations and providing support  **3.1 Set context for lesson (Item 4).**  T.1 Posts, previews, and reviews clear, rigorous, measureable content and language objectives (CLOs).  T.2 Provides rationale that connects content to students’ background experiences, prior knowledge, skills, and/or interests.  T.3 Promotes real-world application that facilitates college and career readiness.  T.4 Clearly defines performance expectations orally and in writing using student-friendly language.  **3.2 Facilitate clear and rigorous learning experiences (Item 5).**  T.5 Provides clear, concise, and comprehensive explanations and representations of content.  T.6 Uses gradual release lesson cadence (I do, we do, you do) to scaffold students’ independent application of learning.  T.7 Aligns learning experiences to objectives.  T.8 Adequately paces learning experiences by attending to student learning cues.  **3.3 Promote rigorous academic talk (Item 6).**  T.9 Promotes high-level thinking by holding students accountable for using academic language, making evidence-based claims, and demonstrating sound reasoning.  T.10 Facilitates inquiry by posing high-level questions and asking students to explain their thinking (e.g., elaborate, clarify, provide examples, build on or challenge ideas, paraphrase, synthesize).  T.11 Encourages students to contribute own perspective, use multiple perspectives, ask probing questions.  **3.4 Make content and language comprehensible (Item 7).**  T.12 Makes strategic use of students’ first language and/or uses materials in students’ home language to increase comprehension of language and content.  T.13 Incorporates a variety of manipulatives and realia that support content learning, language development, and multiple learning styles.  T.14 Makes content comprehensible by incorporating visual representations, explicit vocabulary support, graphic organizers, total physical response, and modeling.  **3.5 Use formal and informal assessment data to monitor student progress toward learning targets (Item 8).**  T.15 Collects data on individual student progress toward meeting the lesson objectives and analyzes data to adjust instruction for individuals and subgroups.  T.16 Engages students in continually assessing their own progress toward lesson objectives.  T.17 Provides frequent, timely, specific, and individualized feedback to students.  T.18 Consistently checks for understanding and adjusts instruction according to evidence of student learning.  **3.6 Differentiate instruction to meet diverse student needs (Item 9).**  T.19 Uses assessment data to differentiate instruction according to students’ levels of language and academic proficiency, learning styles, or interests.  T.20 Implements flexible grouping strategies to meet instructional learning objectives and diverse student needs.  T.21 Presents options for differentiated content, process, or products that allow students to engage in self-directed learning.  T.22 Collaborates with support specialists to develop and apply specific accommodations for individual students based on language needs, IEPs, and other legal requirements. |
| DIMENSION 4: **LEAD** by exemplifying standards of professional practice.  **4.1** **Meet professional standards of practice (Item 10).**  L.1 Adheres to ethical and legal responsibilities for students’ learning, behavior, safety, and/or confidentiality as specified in local, state, and federal statutes.  L.2 Maintains professional demeanor (e.g., business-like appearance, positive relationships & collaboration, appropriate boundaries) in accordance with school, district, and/or university policy.  L.3 Uses standard language in written, verbal, nonverbal, and/or electronic communication.  **4.2** **Demonstrate professional growth and commitment (Item 11).**  L.4 Demonstrates openness and flexibility by taking a learner-stance in interactions with peers, faculty, students, families, and colleagues.  L.5 Analyzes practice for continuous improvement by using feedback and data to set clear and measurable goals to improve instruction and student learning.  L.6 Participates in school, district, and community initiatives (e.g., professional development opportunities, school events, community-based activities). |

^a^Dimension 2 is not included on the FEET observation.
